# Variant analysis of SARS-CoV-2 genomes

**DOI:** 10.2471/BLT.20.253591

**Published:** 2020-06-02

**Authors:** Takahiko Koyama, Daniel Platt, Laxmi Parida

**Affiliations:** a IBM TJ Watson Research Center, 1101 Kitchawan Rd, Yorktown Heights, New York 10598, United States of America.

## Abstract

**Objective:**

To analyse genome variants of severe acute respiratory syndrome coronavirus-2 (SARS-CoV-2).

**Methods:**

Between 1 February and 1 May 2020, we downloaded 10 022 SARS CoV-2 genomes from four databases. The genomes were from infected patients in 68 countries. We identified variants by extracting pairwise alignment to the reference genome NC_045512, using the EMBOSS needle. Nucleotide variants in the coding regions were converted to corresponding encoded amino acid residues. For clade analysis, we used the open source software Bayesian evolutionary analysis by sampling trees, version 2.5.

**Findings:**

We identified 5775 distinct genome variants, including 2969 missense mutations, 1965 synonymous mutations, 484 mutations in the non-coding regions, 142 non-coding deletions, 100 in-frame deletions, 66 non-coding insertions, 36 stop-gained variants, 11 frameshift deletions and two in-frame insertions. The most common variants were the synonymous 3037C > T (6334 samples), P4715L in the open reading frame 1ab (6319 samples) and D614G in the spike protein (6294 samples). We identified six major clades, (that is, basal, D614G, L84S, L3606F, D448del and G392D) and 14 subclades. Regarding the base changes, the C > T mutation was the most common with 1670 distinct variants.

**Conclusion:**

We found that several variants of the SARS-CoV-2 genome exist and that the D614G clade has become the most common variant since December 2019. The evolutionary analysis indicated structured transmission, with the possibility of multiple introductions into the population.

## Résumé

**Objectif:**

Analyser les variantes du génome de coronavirus 2 du syndrome respiratoire aigu sévère (SARS-CoV-2).

**Méthodes:**

Entre le 1^er^ février et le 1^er^ mai 2020, nous avons téléchargé 10 022 génomes de SARS CoV-2 issus de quatre bases de données. Ces génomes provenaient de patients infectés originaires de 68 pays. Nous avons identifié les variantes en procédant à un alignement par paires avec la séquence de référence NC_045512, à l'aide de l'outil EMBOSS Needle. Les variantes de nucléotides dans les régions codantes ont été converties en résidus d'acides aminés codés correspondants. Enfin, pour analyser le clade, nous avons employé un logiciel open source appelé Bayesian Evolutionary Analysis by Sampling Trees, version 2.5.

**Résultats:**

Nous avons détecté 5775 variantes de génome distinctes, dont 2969 mutations faux-sens, 1965 mutations synonymes, 484 mutations dans les régions non codantes, 142 délétions non codantes, 100 délétions sans décalage du cadre de lecture, 66 insertions non codantes, 36 variantes de codon stop, 11 délétions entraînant un décalage du cadre de lecture, et 2 insertions sans décalage du cadre de lecture. Les variantes les plus fréquentes étaient les synonymes 3037C > T (6334 échantillons), P4715L dans le cadre ouvert de lecture 1ab (6319 échantillons) et D614G dans la protéine de spicule (6294 échantillons). Nous avons identifié six clades majeurs (à savoir, de base, D614G, L84S, L3606F, D448del et G392D) et 14 sous-clades. Quant aux changements de base, la mutation C > T était la plus répandue avec 1670 variantes distinctes.

**Conclusion:**

Nous avons constaté qu'il existait de nombreuses variantes du génome de SARS-CoV-2, et que le clade D614G était devenu la variante la plus commune depuis décembre 2019. L'analyse évolutive a indiqué une transmission structurée, avec une possibilité d'introductions multiples au sein de la population.

## Resumen

**Objetivo:**

Analizar las variantes del genoma del coronavirus tipo 2 del síndrome respiratorio agudo grave (SARS-CoV-2).

**Métodos:**

Entre el 1 de febrero y el 1 de mayo de 2020, se registraron 10 022 genomas del CoV-2 del SARS en cuatro bases de datos. Los genomas eran de pacientes infectados ubicados en 68 países. Se identificaron variantes al extraer la alineación por pares del genoma de referencia NC_045512, por medio de EMBOSS Needle. Las variantes de los nucleótidos en las regiones codificantes se convirtieron en los correspondientes residuos de aminoácidos codificados. Para analizar los clados, se utilizó el programa informático de código abierto Bayesian evolutionary analysis by sampling trees, versión 2.5.

**Resultados:**

Se identificaron 5775 variaciones diferentes del genoma, incluidas 2969 mutaciones con cambio de sentido, 1965 mutaciones sinónimas, 484 mutaciones en las regiones no codificantes, 142 supresiones no codificantes, 100 supresiones en la fase, 66 inserciones no codificantes, 36 variaciones de parada prematuras (*stop-gained*), 11 supresiones de desplazamiento de fase y dos inserciones en la fase. Las variaciones más comunes eran las sinónimas 3037C > T (6334 muestras), P4715L en la fase abierta de lectura 1ab (6319 muestras) y D614G en la proteína S (6294 muestras). Se identificaron seis clados principales, (es decir, basal, D614G, L84S, L3606F, D448del y G392D) y 14 subclados. En relación con los cambios de base, la mutación C > T fue la más común con 1670 variaciones diferentes.

**Conclusión:**

Se determinó que existen diversas variaciones del genoma del SARS-CoV-2 y que el clado D614G es la variante más común desde diciembre de 2019. El análisis evolutivo indicó una transmisión estructurada, en la que existe la posibilidad de que se realicen múltiples inserciones en la población.

## ملخص

الغرض تحليل الأشكال المختلفة لجينوم المتلازمة التنفسية الحادة الشديدة المعروفة باسم كورونا فيروس 2 (سارس كوف 2).

الطريقة خلال الفترة ما بين 1 فبراير/شباط، و1 مايو/أيار 2020، قمنا بتنزيل 10022 من جينوم سارس كوف 2 من أربع قواعد بيانات. كانت الجينومات من المرضى حاملي العدوى في 68 دولة. قمنا بتحديد أشكال مختلفة عن طريق استخلاص تنسيق على شكل زوجي من الجينوم المرجعي NC_045512، باستخدام إبرة EMBOSS. تم تحويل الأشكال المختلفة من النيوكليتويد في مناطق الترميز إلى بقايا الحمض الأميني المشفر المقابل. وبالنسبة لتحليل كليد، فقد استخدمنا تحليل بايزان المتطور لبرنامج المصدر المفتوح، عن طريق تفرعات العينات، الإصدار 2.5.

النتائج حددنا 5775 شكلاً مختلفاً ومتميزاً من الجينوم، بما في ذلك 2969 طفرة مُغلطة، و1965 طفرة متشابهة، و484 طفرة في المناطق غير المشفرة، و142 حالة حذف غير مشفرة، و100 حالة حذف في الإطار، و66 إدخال غير مشفر، و36 شكلاً مكتسبًا موقوفاً، و11 حالة حذف لإزاحة الإطار، وعمليتي إدراج داخل الإطار. كانت أكثر الأشكال المختلفة شيوعاً هي المشابه 3037C > T (6334 عينة)، وP4715L في إطار القراءة المفتوحة 1ab (6319 عينة)، وD614G في بروتين الشوكي (6294 عينة). قمنا بتحديد ستة عوامل كليد أساسية (وهي القاعدي، وD614G، وL84S، وL3606FK، D448del، وG392D)، و14 عاملاً فرعياً من كليد. وبخصوص التغييرات القاعدية، فإن طفرة C > T، كانت الأكثر شيوعاً في 1670 شكلاً مختلفاً ومتميزاً.

الاستنتاج لقد اكتشفنا أن هناك العديد من الأشكال المختلفة من جينوم سارس كوف 2، وأن كليد D614G قد أصبح الشكل المختلف الأكثر شيوعاً منذ ديسمبر/كانون أول 2019. أشار التحليل المتطور إلى انتقال منظم، مع إمكانية الظهور المتعدد في السكان.

## 摘要

**目的:**

旨在分析严重急性呼吸综合征冠状病毒 2 (SARS-CoV-2) 的基因组变异体情况。

**方法:**

在 2020 年 2 月 1 日至 5 月 1 日期间，我们从四个数据库下载了 10,022 个严重急性呼吸综合征冠状病毒 2 (SARS-CoV-2) 基因组。这些基因组来自 68 个国家的感染患者。我们通过使用凸出针提取参考基因组 NC_045512 的成对序列比对来确定变异体。编码区的核苷酸变体被转化为相应的编码氨基酸残基。我们使用基于抽样树的开源软件贝叶斯演化分析（2.5 版）进行支系分析。

**结果:**

我们确定了 5775 个不同的基因组变异体，包括 2969 个错义突变、1965 个同义突变、484 个非编码区突变、142 个非编码缺失、100 个框架内缺失、66 个非编码插入、36 个止损变异体、11 个移码缺失和 2 个框架内插入。最常见的变异是同义 3037C > T（6334 个样本）、开放阅读框 1ab 中的 P4715L（6319 个样本）和纤突蛋白中的 D614G（6294 个样本）。我们确定了 6 大主要分支（即，基底、D614G、L84S、L3606F、D448del 和 G392D）和 14 个子分支。在基底变化方面，以 C > T 突变最为常见，共有 1670 个不同的变异体。

**结论:**

我们发现严重急性呼吸综合征冠状病毒 2 (SARS-CoV-2) 基因组存在多种变异体，其中 D614G 支系自 2019 年 12 月以来已成为最常见的变异体。演化分析表明，这是一种结构化传播，有可能多次传入人群中。

## Резюме

**Цель:**

Проанализировать варианты геномов тяжелого острого респираторного синдрома, вызванного коронавирусом‑2 (SARS-CoV-2).

**Методы:**

В период между 1 февраля и 1 мая 2020 года авторы загрузили данные по 10 022 геномам вируса SARS CoV-2 из четырех баз данных. Геномы принадлежали инфицированным пациентам из 68 стран. Авторы идентифицировали варианты, извлекая и попарно сравнивая последовательности с эталонным геномом NC_045512, используя набор инструментов EMBOSS. Варианты нуклеотидной последовательности в кодирующих участках были преобразованы в соответствующие кодируемые аминокислотные остатки. Для анализа клад использовалось программное обеспечение с открытым кодом для байесовского эволюционного анализа деревьев выборки, версия 2.5.

**Результаты:**

Было идентифицировано 5775 четких вариантов генома, в том числе 2969 миссенс-мутаций, 1965 синонимичных мутаций, 484 мутации в некодирующих участках, 142 некодирующие делеции, 100 делеций внутри рамки считывания, 66 некодирующих вставок, 36 вариантов изменения последовательности ДНК с новым стоп-кодоном, 11 делеций со сдвигом рамки и две вставки внутри рамки считывания. Чаще всего встречались синонимичная замена 3037C > T (6334 образца), P4715L в открытой рамке считывания 1ab (6319 образцов) и D614G в белке «шипа» (6294 образца). Было выявлено шесть основных клад (базовая, D614G, L84S, L3606F, D448del и G392D) и 14 субклад. Что касается замены оснований, наиболее частой была мутация с заменой цитозина на тимин (C>T), которая встречалась в 1670 вариантах.

**Вывод:**

Авторы обнаружили существование нескольких вариантов генома SARS-CoV-2 и выяснили, что с декабря 2019 года наиболее распространенным вариантом является клада D614G. Эволюционный анализ продемонстрировал структурированную передачу генетических данных с возможностью многократной интродукции в популяцию.

## Introduction

In late 2019, several people in Wuhan, China, were presenting with severe pneumonia at the hospitals. As the number of patients rapidly increased, the Chinese government decided on 23 January 2020 to lock down the city to contain the virus. Unfortunately, the virus had already spread across China and throughout the world. The World Health Organization (WHO) officially declared the outbreak a pandemic on March 11, 2020. As of 23 May 2020, over 5 million cases worldwide had been reported to WHO and the death toll has exceeded 330 000.^1^

Researchers isolated the virus causing the pneumonia in December 2019 and found it to be a strain of *β*-coronavirus (CoV). The virus showed a high nucleotide sequence homology with two severe acute respiratory syndrome (SARS)-like bat coronaviruses, bat-SL-CoVZC45 and bat-SL-CoVZXC21 (88% homology) and with SARS-CoV (79.5% homology), while only 50% homology with the Middle East respiratory syndrome coronavirus (MERS) CoV.^2^^,^^3^ The virus, now named SARS-CoV-2, contains a single positive stranded RNA (ribonucleic acid) of 30 kilobases, which encodes for 10 genes.^4^ Researchers have shown that the virus can enter cells by binding the angiotensin-converting enzyme 2 (ACE2), through its receptor binding domain in the spike protein.^5^

The virus causes the coronavirus disease 2019 (COVID-19), with common symptoms such as fever, cough, shortness of breath and fatigue.^6^^,^^7^ Early data indicated that about 20% of patients develop severe COVID-19 requiring hospitalization, including 5% who are admitted to the intensive care unit.^8^ Initial estimates of the case fatality rates were from 3.4% to 6.6% which is lower than that of SARS or MERS, 9.6% and 34.3% respectively.^9^^–^^11^ The mortality from COVID-19 is higher in people older than 65 years and in people with underlying comorbidities, such as chronic lung disease, serious heart conditions, high blood pressure, obesity and diabetes.^12^^–^^14^

Community transmission of the virus, as well as anti-viral treatments, can engender novel mutations in the virus, potentially resulting in more virulent strains with higher mortality rates or emergence of strains resistant to treatment.^15^ Therefore, systematic tracking of demographic and clinical patient information, as well as strain information is indispensable to effectively combat COVID-19. 

Here we analysed the SARS-CoV-2 genome from 10 022 samples to understand the variability in the viral genome landscape and to identify emerging clades.

## Methods

In total, we downloaded 15 755 genome sequences from the following databases: the Chinese National Microbiology Data Center on 1 February 2020; the Chinese National Genomics Data Center Genome Warehouse on 4 February 2020; GISAID^16^ on 1 May 2020 and GenBank on 1 May 2020. We removed redundant sequences with the China National Center for Bioinformation annotations. To reduce the number of false positive variants, we removed sequences with more than 50 ambiguous bases.

For this study, we used the sequence of established SARS-CoV-2 reference genome, NC_045512.^17^ This genome was sequenced in December 2019. Each sample was first aligned to the reference genome in a pairwise manner using EMBOSS needle (Hinxton, Cambridge, England), with a default gap penalty of 10 and extension penalty of 0.5.^18^ Then, we developed a custom script in Python (Python Software Foundation, Wilmington, United States of America) to extract the differences between the genome variants and the reference genome. Nucleotide variants in the coding regions were converted to corresponding encoded amino acid residues. For the open reading frame 1 (ORF1), we used the protein coordinates from YP_009724389.1^19^ for translation. Finally, we carefully investigated stop-gained and frameshift variants causing deletions and insertions to detect potential artefacts caused by undetermined or ambiguous bases. The results are provided in a list of variants (available in the data repository).^20^

Using the identified recurrent variants, we performed hierarchical clustering in SciPy library, Python, to identify clades. First, a binary matrix of samples and distinct variants was created. Then, we did hierarchical clustering using the Ward’s method^21^ in SciPy library.^22^

We investigated the mutation patterns of SARS-CoV-2 to find potential causes of mutations, by looking at the changes in bases. Since coronavirus genomes are positive sense, single stranded RNA, we did not combine C > T with G > A mutations.

The spike protein is a key protein for SARS-CoV-2 viral entry and a target for vaccine development. We, therefore, wanted to find amino acid conservation between other coronavirus sequences in the spike protein. We used the basic local alignment search tool BLAST (National Center for Biotechnology Information [NCBI], Bethesda, United States)^23^ followed by the constraint-based multiple alignment tool COBALT (NCBI, Bethesda, United States).^24^ We carefully investigated mutations within the receptor binding domain and predicted B-cell epitopes.^25^^,^^26^ The mutations were further analysed to identify cross species conservation and to understand the nature of amino acid changes. We visualized the aligned sequence using the open source software alv.^27^

For the phylogenetic analysis, we used the open source software Bayesian evolutionary analysis by sampling trees (BEAST), version 2.5.^28^ BEAST uses a Bayesian Monte-Carlo algorithm generating a distribution of likely phylogenies given a set of priors, based on the probabilities of those tree configurations determined from the viral genomes. This analysis presents a different view than the variant analysis described above and is an independent test of the structure that individual haplogroup markers identify. First, we aligned sequences to NC_045512, using the multiple sequence alignment software, MAFFT.^29^ Subsequently, we adjusted for length and sequencing errors, by truncating the bases in the 5’-UTR and 3’-UTR, without losing key sites. We excluded sequences showing a variability higher than 30 bases. For an optimal output of the phylogenetic tree, we randomly selected a subset of 2000 samples by using a random number generator in Python. We ran BEAST using sample collection dates with the Hasegawa-Kishino-Yano mutation model,^30^ with the strict clock mode. Finally, we estimated the mutation rate and median tree height from the resulting BEAST trees.

## Results

In total, we analysed 10 022 SARS CoV-2 genomes (sequences are available from the data repository)^20^ from 68 countries. Most genomes came from the United States of America (3543 samples), followed by the United Kingdom of Great Britain and Northern Ireland (1987 samples) and Australia (760 samples; Box 1). We detected in total 65776 variants with 5775 distinct variants. The 5775 distinct variants consist of 2969 missense mutations, 1965 synonymous mutations, 484 mutations in the non-coding regions, 142 non-coding deletions, 100 in-frame deletions, 66 non-coding insertions, 36 stop-gained variants, 11 frameshift deletions and two in-frame insertions (Table 1).

Box 1 **Number of samples of severe acute respiratory syndrome coronavirus 2 from each country or territory included in sequence analysis, 2019–2020**

United States 3543 samples; United Kingdom 1987 samples; Australia 760 samples; Iceland 461 samples; Netherlands 402 samples; China 342 samples; Belgium 335 samples; Denmark 260 samples; France 218 samples; Spain 148 samples; Russian Federation 141 samples; Canada 117 samples; Luxembourg 112 samples; Sweden 107 samples; Portugal 96 samples; Japan 95 samples; Taiwan, China 85 samples; Singapore 71 samples; Germany 61 samples; Switzerland 55 samples; India 51 samples; Italy 44 samples; Brazil 43 samples; China, Hong Kong Special Administrative Region 43 samples; Greece 41 samples; Republic of Korea 36 samples; Czechia 34 samples; Turkey 25 samples; Argentina 24 samples; Finland 24 samples; Thailand 22 samples; Jordan 20 samples; Norway 18 samples; Austria 15 samples; Senegal 15 samples; Democratic Republic of the Congo 14 samples; Georgia 12 samples; Malaysia 12 samples; Mexico 11 samples; Ireland 10 samples; Latvia 10 samples; Viet Nam 10 samples; Poland 9 samples; Sri Lanka 8 samples; Chile 7 samples; Kuwait 7 samples; New Zealand 6 samples; Costa Rica 5 samples; South Africa 5 samples; Estonia 4 samples; Slovakia 4 samples; Slovenia 4 samples; Algeria 3 samples; Gambia 3 samples; Hungary 3 samples; Israel 3 samples; Pakistan 3 samples; Saudi Arabia 3 samples; Belarus 2 samples; Nepal 2 samples; Peru 2 samples; Philippines 2 samples; Qatar 2 samples; Cambodia 1 sample; Colombia 1 sample; Egypt 1 sample; Iran (Islamic Republic of) 1 sample; and Lithuania 1 sample.

**Table 1:** Number of gene variants in SARS-CoV-2 genomes,2019–2020

Genome segment^a^	Missense mutation	Synonymous mutation		Non-coding region				In-frame			Frameshift deletion	Stop-gained	Total
				Mutation	Deletion	Insertion		Deletion	Insertion				
*ORF1ab*	1905	1344		0	0	0		57	2		7	13	3328
*S*	394	260		0	0	0		27	0		0	6	687
*ORF3a*	169	71		0	0	0		5	0		1	1	247
*E*	27	15		0	0	0		1	0		0	0	43
*M*	53	71		0	0	0		0	0		0	0	124
*ORF6*	28	11		0	0	0		2	0		0	2	43
*ORF7*	59	29		0	0	0		1	0		2	6	97
*ORF8*	68	26		0	0	0		1	0		0	7	102
*ORF10*	20	12		0	0	0		0	0		1	1	34
*N*	246	126		0	0	0		6	0		0	0	378
Intergenic	0	0		0	7	2		0	0		0	0	9
5’-UTR	0	0		260	50	37		0	0		0	0	347
3’-UTR	0	0		224	85	27		0	0		0	0	336
**Total**	**2969**	**1965**		**484**	**142**	**66**		**100**	**2**		**11**	**36**	**5775**

E: envelope protein; M: membrane glycoprotein; N: nucleocapsid phosphoprotein; ORF: open reading frame; S: spike glycoprotein; SARS-CoV-2: severe acute respiratory syndrome coronavirus 2; UTR: untranslated region.

^a^ Genes are in italics.

Note: We compared 10 022 genomes to the NC_045512 genome sequence.^17^

Of the 2969 missense variants, 1905 variants are found in ORF1ab, which is the longest ORF occupying two thirds of the entire genome. ORF1ab is transcribed into a multiprotein and subsequently cleaved into 16 nonstructural proteins (NSPs). Of these proteins, NSP3 has the largest number of missense variants among ORF1ab proteins. Of the NSP3 missense variants, A58T was the most common (159 samples) followed by P153L (101 samples; Table 2). We also detected mutations in the nonstructural protein RNA-dependent RNA polymerase (RdRp), such as P323L (6319 samples). Deletions are also common in 3′-5′exonuclease (11 deletions) including those resulting in frameshifts. A comprehensive list of variants is available in data repository.^20^

**Table 2:** Number of variants in the open reading frame 1ab of SARS-CoV-2 genomes, by final cleaved protein, 2019–2020

Final protein^a^	Missense mutation	Synonymous mutation		Non-coding region				In-frame			Frameshift deletion	Stop-gained	Total
				Mutation	Deletion	Insertion		Deletion	Insertion				
NSP1	64	45		0	0	0		13	0		1	0	123
NSP2	237	130		0	0	0		5	0		0	0	372
NSP3	547	349		0	0	0		16	0		2	3	917
NSP4	116	113		0	0	0		1	0		0	1	232
3CLPro	67	54		0	0	0		0	0		0	0	121
NSP6	82	67		0	0	0		4	1		2	0	156
NSP7	27	21		0	0	0		0	0		0	0	48
NSP8	60	25		0	0	0		1	0		0	1	87
NSP9	29	22		0	0	0		0	0		0	1	52
NSP10	25	25		0	0	0		0	0		0	2	52
RdRp	194	157		0	0	0		2	0		1	3	357
Helicase	148	101		0	0	0		0	0		0	0	249
ExoN	141	118		0	0	0		11	0		1	2	273
endoRNase	92	67		0	0	0		3	0		0	0	162
OMT	76	50		0	0	0		1	1		0	0	128
**Total**	**1905**	**1344**		**0**	**0**	**0**		**57**	**2**		**7**	**13**	**3329**

3CLPro: 3C like protease; ExoN: 3-’5′ exonuclease; NSP: non-structural protein; OMT: O-methyltransferase; RdRp: RNA-dependent RNA polymerase; SARS-CoV-2: severe acute respiratory syndrome coronavirus 2.

^a^ The *open reading frame 1ab* gene codes for a polyprotein, which a viral protease cleaves in to several protein after translation.

Note: We compared 10 022 genomes to the NC_045512 genome sequence.^17^

Variants with recurrence over 100 samples are shown in Table 3. The most common variants were the synonymous variant 3037C > T (6334 samples), ORF1ab P4715L (RdRp P323L; 6319 samples) and SD614G (6294 samples). They occur simultaneously in over 3000 samples, mainly from Europe and the United States. Other variants including ORF3a Q57H (2893 samples), ORF1ab T265I (NSP3 T85I; 2442 samples), ORF8 L84S (1669 samples), N203_204delinsKR (1573 samples), ORF1ab L3606F (NSP6 L37F; 1070 samples) were the key variants for identifying clades.

**Table 3:** Variants of SARS-CoV-2 genomes observed in more than 100 samples, 2019–2020

Genomic change	Type of mutation	Gene/protein	Amino acid change	No. of samples
**3037C > T**	Synonymous	*ORF1ab/*NSP3	F924F/F106F	6334
**14408C > T**	Missense	*ORF1ab/*RdRp	P4715L/P323L	6319
**23403A > G**	Missense	*S*	D614G	6294
**241C > T**	Non-coding	*5’-UTR*	NA	5928
**25563G > T**	Missense	*ORF3a*	Q57H	2893
**1059C > T**	Missense	*ORF1ab*/NSP2	T265I/T85I	2442
**28144T > C**	Missense	*ORF8*	L84S	1669
**8782C > T**	Synonymous	*ORF1ab*/NSP4	S2839S/S76S	1598
**28881_28883delinsAAC**	Missense	*N*	203_204delinsKR	1573
**18060C > T**	Synonymous	*ORF1ab*/ExoN	L5932L/L7L	1178
**17858A > G**	Missense	*ORF1ab*/helicase	Y5865C/Y541C	1166
**17747C > T**	Missense	*ORF1ab*/helicase	P5828L/P504L	1147
**11083G > T**	Missense	*ORF1ab*/NSP6	L3606F/L37F	1070
**14805C > T**	Synonymous	*ORF1ab*/RdRp	Y4847Y/Y455Y	844
**26144G > T**	Missense	*ORF3a*	G251V	769
**20268A > G**	Synonymous	*ORF1ab*/endoRNase	L6668L/L216L	452
**17247T > C**	Synonymous	*ORF1ab*/helicase	R5661R/R337R	325
**2558C > T**	Missense	*ORF1ab*/NSP2	P765S/P585S	274
**15324C > T**	Synonymous	*ORF1ab*/RdRp	N5020N/N628N	267
**1605_1607del**	In-frame deletion	*ORF1ab*/NSP2	D448del/D268del	250
**18877C > T**	Synonymous	*ORF1ab*/ExoN	L6205L/L280L	234
**2480A > G**	Missense	*ORF1ab*/NSP2	I739V/I559V	232
**27046C > T**	Missense	*M*	T175M	221
**11916C > T**	Missense	*ORF1ab*/NSP7	S3884L/S25L	185
**2416C > T**	Synonymous	*ORF1ab*/NSP2	Y717Y/Y537Y	170
**1440G > A**	Missense	*ORF1ab*/NSP2	G392D/G212D	164
**27964C > T**	Missense	*ORF8*	S24L	164
**36C > T**	Non-coding	*5’-UTR*	NA	163
**2891G > A**	Missense	*ORF1ab*/NSP3	A876T/A58T	159
**28854C > T**	Missense	*N*	S194L	155
**1397G > A**	Missense	*ORF1ab*/NSP2	V378I/V198I	139
**28657C > T**	Synonymous	*N*	D128D	139
**28688T > C**	Synonymous	*N*	L139L	138
**18998C > T**	Missense	*ORF1ab*/ExoN	A6245V/A320V	137
**28311C > T**	Missense	*N*	P13L	136
**28863C > T**	Missense	*N*	S197L	136
**9477T > A**	Missense	*ORF1ab*/NSP4	F3071Y/F308Y	136
**25979G > T**	Missense	*ORF3a*	G196V	132
**29742G > T**	Non-coding	*3’-UTR*	NA	131
**25429G > T**	Missense	*ORF3a*	V13L	128
**24034C > T**	Synonymous	*S*	N824N	118
**29870C > A**	Non-coding	*3’-UTR*	NA	115
**28077G > C**	Missense	*ORF8*	V62L	113
**26729T > C**	Synonymous	*M*	A69A	106
**27_37del**	Non-coding deletion	*5’-UTR*	NA	106
**19_24del**	Non-coding deletion	*5’-UTR*	NA	105
**514T > C**	Synonymous	*ORF1ab*/NSP1	H83H/H83H	105
**23731C > T**	Synonymous	*S*	T723T	102
**3177C > T**	Missense	*ORF1ab*/NSP3	P971L/T1198K	101

del: deletion; delins: deletion–insertion; ExoN: 3’-5′ exonuclease; NSP: non-structural protein; M: membrane glycoprotein; N: nucleocapsid phosphoprotein; NA: not applicable; ORF: open reading frame; RdRp: RNA-dependent RNA polymerase; SARS-CoV-2: severe acute respiratory syndrome coronavirus 2; S: spike glycoprotein; UTR: untranslated region.

Note: We compared 10 022 genomes to the NC_045512 genome sequence.^17^

**Fig. 1:**
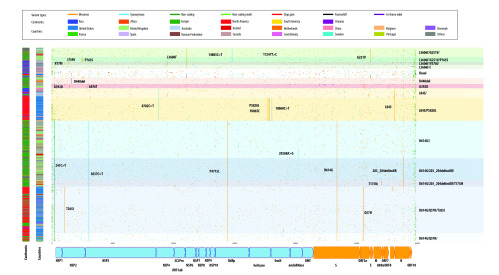
A graphical representation of variants found in SARS-CoV-2 genomes, 2019–2020

We identified six major clades with 14 subclades (Fig. 1 and Table 4). The largest clade is D614G clade with five subclades. Most samples in the D614G clade also display the non-coding variant 241C > T, the synonymous variant 3037C > T and ORF1ab P4715L. Within D614G clade, D614G/Q57H/T265I subclade forms the largest subclade with 2391 samples. The second largest major clade is L84S clade, which was observed among travellers from Wuhan in the early days of the outbreak, and the clade consists of 1662 samples with 2 subclades. The L84S/P5828L/ subclade is predominantly observed in the United States. Among the L3606F subclades, L3606F/G251V/ forms the largest group with 419 samples. G251V frequently appears in samples from the United Kingdom (329 samples), Australia (95 samples), the United States (80 samples) and Iceland (76 samples). However, the basal clade now accounts only for a small fraction of genomes (670 samples mainly from China). The remaining two clades D448del and G392D are small and they are without any significant subclades at this point.

3CLPro: 3C like protease; del: deletion; delins: deletion–insertion; E: envelope protein; ExoN: 3’-5’ exonuclease; M: membrane glycoprotein; N: nucleocapsid phosphoprotein; NA: not applicable; NSP: non-structural protein; OMT: O-methyltransferase; ORF: open reading frame; RdRp: RNA-dependent RNA polymerase; SARS-CoV-2: severe acute respiratory syndrome coronavirus 2; S: spike glycoprotein; UTR: untranslated region.

Notes: Variants are coloured depending on the type of mutations (missense, synonymous, non-coding, stop-gained, and frameshift). Major variants are annotated, and clades are indicated by horizontal colour stripes. Continents and countries from where samples originated are shown in the bars on the left. The gene structure is displayed at the bottom. Countries with samples in the African continent: Algeria, Democratic Republic of the Congo, Egypt, Gambia, Senegal and South Africa; Asian continent: Cambodia, China, Georgia, India, Iran (Islamic Republic of), Israel, Japan, Jordan, Kuwait, Malaysia, Nepal, Pakistan, Philippines, Qatar, Republic of Korea, Saudi Arabia, Singapore, Sri Lanka, Thailand and Viet Nam; European continent: Austria, Belarus, Belgium, Czechia, Denmark, Estonia, Finland, France, Germany, Greece, Hungary, Iceland, Ireland, Italy, Latvia, Lithuania, Luxembourg, Netherlands, Norway, Poland, Portugal, Slovakia, Slovenia, Spain, Sweden, Switzerland, Russian Federation, Turkey and United Kingdom; North America: Canada, Mexico and United States; Oceania; Australia and New Zealand; South America: Argentina, Brazil, Chile, Colombia, Costa Rica and Peru.

**Table 4:** Major clades of SARS-CoV-2 genomes, 2019–2020

Clade/sublevel 1/sublevel 2	First observation of strain			No. of samples
	Date	Accession no.	Country	
**Basal^a^**	Dec 2019	MN90894	China	670
**D614G//**	24 Jan 2020	EPI_ISL_422425	China	1889
D614G/Q57H/	26 Feb 2020	EPI_ISL_418219	France	469
D614G/Q57H/T265I	21 Feb 2020	EPI_ISL_418218	France	2391
D614G/203_204delinsKR/	25 Feb 2020	EPI_ISL_412912	Germany	1330
D614G/203_204delinsKR/T175M	1 Mar 2020	EPI_ISL_413647 and EPI_ISL_417688	Portugal and Iceland	215
**L84S//**	30 Dec 2019	MT291826	China	525
L84S/P5828L	20 Feb 2020	EPI_ISL_413456	United States	1137
**L3606F//**	18 Jan 2020	EPI_ISL_408481	China	182
L3606F/V378I/	18 Jan 2020	EPI_ISL_412981	China	127
L3606F/G251V/	29 Jan 2020	EPI_ISL_412974	Italy	419
L3606F/G251V/P765S	20 Feb 2020	EPI_ISL_415128	Brazil	260
**D448del//**	8 Feb 2020	EPI_ISL_410486,	France	248
**G392D//**	25 Feb 2020	EPI_ISL_414497	Germany	160

Del: deletion; delins: deletion–insertion; SARS-CoV-2: severe acute respiratory syndrome coronavirus 2.

^a^ The reference genome (NC_045512)^17^ used in this study belongs to the basal clade.

All non-coding deletions are either located within 3’-UTR, 5’-UTR or intergenic regions. Of the in-frame deletions, ORF1 D448del stands out with 250 samples. In contrast, we only detected two distinct in-frame insertions in our data set. We also detected 11 frameshift deletions and 36 stop-gained variants. The recurrent stop-gained variant Y4379* (NSP10 Y126*) is found in 51 samples in the D614G clade. NSP10 Y126* is located only 13 residues upstream of the stop codon; therefore, a truncation may not significantly affect function of the protein. Most of frameshift variants in ORF1ab do not recur except for S135fs (three samples) and L3606fs (two samples). Although frameshift variants are considered deleterious, for instance, S135fs (more precisely S135Rfs*9) caused by 670_671del, ORF1ab is truncated at residue 143 before NSP2 and translation might resume from the methionine at residue 174 near the end of NSP1. Other notable recurrent frameshift variants include ORF3a V256fs and ORF7 I103fs.

**Fig. 2:**
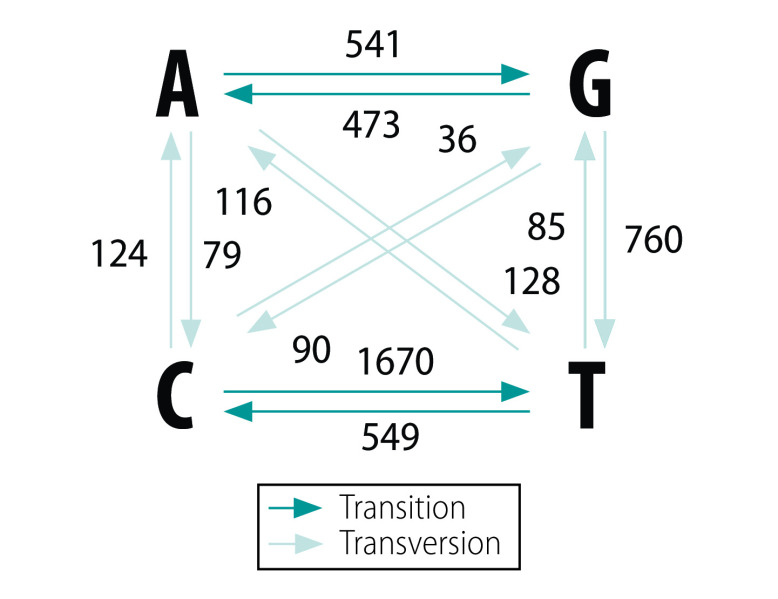
Base pair changes observed in SARS-CoV-2 genomes, 2019–2020

The most common base change is C > T (Fig. 2). As expected,^31^ we observed a strong bias in transition versus transversion ratio (7:3). C > T transitions might be intervened by cytosine deaminases. Surprisingly, G > T transversions, likely introduced by oxo-guanine from reactive oxygen species,^32^ were also frequently observed.

SARS-CoV-2: severe acute respiratory syndrome coronavirus 2.

Notes: The data come from 10 022 analysed genomes. The arrows indicate how bases are changed. Numbers next to the arrows indicate the number of distinct variants with those types of changes.

**Fig. 3:**
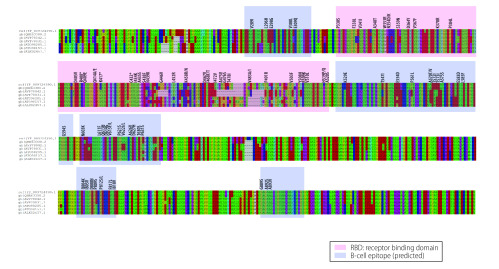
Annotation of SARS-CO-2 variants in the alignment of the amino acid sequence of the spike protein from several coronaviruses, 2019–2020

Assessing variants in the spike protein revealed 427 distinct non-synonymous variants with many variants located within the receptor binding domain and B-cell epitopes (Fig. 3). Among the variants in the receptor binding domain, V483A (26 samples), G476S (9 samples) and V367F (12 samples) are highly recurrent.

SARS-CoV-2: severe acute respiratory syndrome coronavirus 2.

Notes: We aligned amino acids sequences of the Spike protein from SARS-CoV-2 (YP_009724390.1), Bat CoV RaTG13 (QHR63300.2), Bat SARS-like CoVs(AVP78042.1, AVP78031.1, ATO98205.1 and ATO98157.1) and SARS-like CoV WIV16 (ALK02457.1). Receptor binding domain and predicted B-cell epitopes are highlighted and the variants we identified in those segments are marked. The colour coding for the amino acids is by amino acid characteristic.

**Fig. 4:**
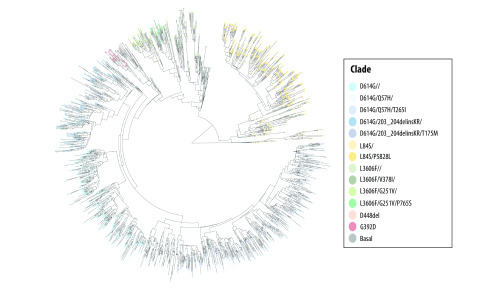
Phylogenetic tree for the SARS-CoV-2 genomes, 2019–2020

Fig. 4 shows the consensus tree from the phylogenetic analysis. The tree has a coalescence centre with exponential expansion identified by haplotype markers. The colour mapped phylogenies largely support the 14 identified subclades. We note that substantial numbers of samples from the United States show affinity with European lineages rather than those directly derived from East Asia. Except for the earliest cases, European clades dominate even in samples from western states in the United States. Further, European samples tend to associate with lineages that expanded through Australia.

SARS-CoV-2: severe acute respiratory syndrome coronavirus 2.

Notes: Each sample is coloured with corresponding subclade. We used the Bayesian evolutionary analysis by sampling trees software.^28^

Estimation of mutation rate showed a median of 1.12 × 10^−3^ mutations per site-year (95% confidence interval, CI: 9.86 × 10^−4^ to 1.85 × 10^−4^). The median tree height was 5.1 months (95% CI: 4.8 to 5.52).

## Discussion

Here we show the evolution of the SARS-Co-2 genome as it has spread across the world. Although, our methods do not allow us to investigate whether the mutations observed led to a loss or gain of function, we can speculate on the implications of viral function of these mutations.

The most common clade identified was the D614G variant, which is located in a B-cell epitope with a highly immunodominant region and may therefore affect vaccine effectiveness.^33^ Although amino acids are quite conserved in this epitope, we identified 14 other variants besides D614G. Almost all strains with D614G mutation also have a mutation in the protein responsible for replication (ORF1ab P4715L; RdRp P323L), which might affect replication speed of the virus. This protein is the target of the anti-viral drugs, remdesivir and favipiravir, and the susceptibility for mutations suggests that treatment resistive strains may emerge quickly. Mutations in the receptor binding domain of the spike protein suggest that these variants are unlikely to reduce binding affinity with ACE2, since that would decrease the fitness of the virus. V483A and G476S are primarily observed in samples from the United States, whereas V367F is found in samples from China, Hong Kong Special Administrative Region, France and the Netherlands. The V367F and D364Y variants have been reported to enhance the structural stability of the spike protein facilitating more efficient binding to the ACE2 receptor.^34^ In summary, structural and functional changes concomitant with spike protein mutations should be meticulously studied during therapy design and development.

We detected several non-recurring frameshift variants, which can be sequencing artefacts. The frameshift at Y3 in ORF10, although only detected in one sample, might not be essential for survival of the new coronavirus, since ORF10, a short 38-residue peptide, is not homologous with other proteins in the NCBI repository.

The phylogenetic analysis suggest population structuring in the evolution of SARS-CoV-2. The analysis provides an independent test of the major clades we identified, as well as the geographic expansions of the variants. While the earliest samples from the United Stated appear to be derived from China, belonging either to basal or L84S clades, the European clades, such as D614G/Q57H, tend to associate with most of the subsequent increase in infected people in the United States. D614G was first observed in late January in China and became the largest clade in three months. The mutation rate of 1.12 × 10^−3^ mutations per site-year is similar to 0.80 × 10^−3^ to 2.38 × 10^−3^ mutations per site-year reported for SARS-CoV-1.^35^

The rapid increase of infected people will provide more genome samples that could offer further insights to the viral dissemination, particularly the possibility of at least two zoonotic transmissions of SARS-CoV-2 into the human population. An understanding of the biological reservoirs carrying coronaviruses and the modalities of contact with human population through trade, travel or recreation will be important to understand future risks for novel infections. Further, populations may be infected or even re-infected via multiple travel routes.

The number of people with confirmed COVID-19 has rapidly increased over the last five months with no sign of decline in the near future. The fight against COVID-19 will be long, until vaccines and other effective therapies are developed. To facilitate rapid therapeutic development, clinicopathological, genomic and other societal information must be shared with researchers, physicians and public health officials. Given the evolving nature of the SARS-CoV-2 genome, drug and vaccine developers should continue to be vigilant for emergence of new variants or sub-strains of the virus.
